# Automatic Classification of Colour Fundus Images for Prediction Eye Disease Types Based on Hybrid Features

**DOI:** 10.3390/diagnostics13101706

**Published:** 2023-05-11

**Authors:** Ahlam Shamsan, Ebrahim Mohammed Senan, Hamzeh Salameh Ahmad Shatnawi

**Affiliations:** 1 Computer Department, Applied College, Najran University, Najran 66462, Saudi Arabia; hsshatnawi@nu.edu.sa; 2 Department of Artificial Intelligence, Faculty of Computer Science and Information Technology, Alrazi University, Sana’a, Yemen

**Keywords:** DenseNet-121, MobileNet, ANN, eye diseases, PCA, handcrafted features

## Abstract

Early detection of eye diseases is the only solution to receive timely treatment and prevent blindness. Colour fundus photography (CFP) is an effective fundus examination technique. Because of the similarity in the symptoms of eye diseases in the early stages and the difficulty in distinguishing between the type of disease, there is a need for computer-assisted automated diagnostic techniques. This study focuses on classifying an eye disease dataset using hybrid techniques based on feature extraction with fusion methods. Three strategies were designed to classify CFP images for the diagnosis of eye disease. The first method is to classify an eye disease dataset using an Artificial Neural Network (ANN) with features from the MobileNet and DenseNet121 models separately after reducing the high dimensionality and repetitive features using Principal Component Analysis (PCA). The second method is to classify the eye disease dataset using an ANN on the basis of fused features from the MobileNet and DenseNet121 models before and after reducing features. The third method is to classify the eye disease dataset using ANN based on the fused features from the MobileNet and DenseNet121 models separately with handcrafted features. Based on the fused MobileNet and handcrafted features, the ANN attained an AUC of 99.23%, an accuracy of 98.5%, a precision of 98.45%, a specificity of 99.4%, and a sensitivity of 98.75%.

## 1. Introduction

The retina is made up of a light layer that lines the eye’s inner back. It works to convert the light falling into the eye into nerve signals, which are sent to the optical cortex of the brain for processing and object recognition [1]. Eye diseases that are not diagnosed early damage the retina and cause irreversible vision loss. Therefore, early diagnosis is necessary to receive appropriate treatment [2,3]. There are multiple techniques for analysing retinal images to detect eye diseases, which are widely used, namely CFP and optical tomography (OCT) [4]. The OCT method produces images to measure the thickness of the retina to diagnose an eye condition, which is an expensive technique. The CFP method produces images of the inner surfaces of the eyes to identify retinal disorders. Both methods are effective for diagnosing eye diseases, but CFP is more effective, less expensive, and non-invasive for detecting eye diseases. It is recommended for all adults, especially adults, to undergo continuous examinations using the CFP method [5]. Ophthalmologists use fundus images to analyse them and detect eye diseases such as cataracts, diabetic retinopathy, glaucoma, and other eye diseases [6]. Some factors cause cataracts, such as ageing, immune abnormalities, heredity, trauma, metabolism, radiation, and metabolic disorders of the lens, which lead to opacity. Consequently, the person suffers from a cataract due to protein denaturation of the lens, preventing light from reaching the retina [7]. Retinopathy is due to high blood glucose levels in diabetic patients. High blood glucose leads to the leakage of fluids and blood into the retina’s blood vessels, which leads to blindness [8]. Retinopathy goes through many stages that can be treated if diagnosed early. Glaucoma is one of the serious eye diseases, and its signs include optic disc atrophy, optical domain faults, depression, and vision loss. High blood pressure and decreased supply through the optic nerve led to glaucoma [9]. Unfortunately, the initial symptoms of most eye diseases are similar, which poses a challenge for ophthalmologists to make an accurate and effective diagnosis in the early stages. In addition, the manual diagnosis of large amounts of images generated with CFP is tedious and time-consuming. Furthermore, developing countries do not have sufficient ophthalmologists to conduct a manual diagnosis. Thus, there is an urgent need for automatic diagnosis to improve diagnostic accuracy, reduce the burden on ophthalmologists, and support their decisions. Due to the increasing number of patients with eye diseases and the lack of ophthalmologists, computer-aided diagnosis is an effective method for automatic disease detection. The academic and industry communities have dedicated their efforts to designing automatic techniques, particularly CNN models, for analysing biomedical images. For eye diseases, CNN models demonstrate their high performance in disease classification. CNN aims to help doctors and experts by supporting their decisions, reducing the burden, and reducing the time to classify the disease. Early diagnosis of eye diseases is crucial to avoid disease progression to advanced stages that lead to blindness. Much of the research on fundus image analysis is limited to a single disease and early diagnosis of its severity. This study aims to develop automated models capable of analysing fundus images to classify several eye diseases. There is a similarity in the clinical and vital signs of eye diseases in their early stages; thus, the CNN models worked on extracting and integrating fine and hidden features. The handcrafted features were obtained and serially combined with the CNN features.

The major contributions of this work are:

- Two overlapping filters improve all CFP pictures to enhance images and improve the contrast in the regions of interest.
- Combining the features of MobileNet and DenseNet121 models before and after dimension reduction.
- Extracting texture, colour, and shape features using Gray Level Co-occurrence Matrix (GLCM), Fuzzy Colour Histogram (FCH), Local Binary Pattern (LBP), and Discrete Wavelet Transform (DWT) methods and then combining them into so-called handcrafted features.
- Combining the MobileNet-handcrafted and DenseNet121-handcrafted features.

## 2. Related Work

Junjun et al. [10] proposed DCNet to classify multi-marker eye diseases. The network consists of three consecutive units: the backbone for extracting features from fundus images, the correlation of features with a spatial correlation unit, and the classification unit. Neha et al. [11] proposed four CNN models with different optimisers to classify multi-class fundus images for an Ocular Disease Intelligent Recognition (ODIR) dataset that contained changes in anatomical structures such as optic disc, macula, and blood vessels. VGG16 with SGD optimisers achieved better results for fundus image classification than other models. Xiong et al. [12] presented a deep learning model with a mixture loss function to analyse fundus images to detect eye diseases. A combination of the loss function and focal loss robustness in a deep learning model was introduced to improve the classification of the eye disease dataset. Kai et al. [13] presented a method for the segmentation of retinal vasculature from CFP images according to the CNN model. A probability map is produced based on the CNN model with a loss function. The CNN model was built by collecting feature maps to extract accurate information about retinal vessels. Clement et al. [14] proposed a system to train the Messidor dataset on a convolutional structure and reinforce it with supervised learning. The red and bright lesions are segmented simultaneously with the detection of lesions. The system generates slices containing red and bright lesions, which the system verifies at the pixel level. The system achieved an Area Under Curve (AUC) of 83.9%. Rahul et al. [15] proposed CNN and support vector machine (SVM) to micro-read fundus images for cataract detection. The number of images in the dataset increased from the original images, and features were extracted. CNN attained an accuracy of 87.08%, while SVM attained an accuracy of 87.5%. Masum et al. [16] proposed CataractNet to analyse fundus images for cataract detection. The computational cost of CataractNet is reduced by adjusting the loss function with fewer parameters and smaller kernels. Yih-Chung et al. [17] developed a deep-learning model based on CFP images for cataract detection. The model achieved an AUC of 96.6% compared to 91.6% by four ophthalmologists. Jiewei et al. [18] developed two strategies based on the Hough transform and Faster CNN to improve cataract detection. Refinements in lens opacity intensity scores and location were made using Grad-Cam and t-distributed stochastic neighbour embedding (t-SNE) methods to highlight the region of interest and select high-level features. Yaroub et al. [19] proposed a lightweight MobileNet-V2 model for cataract classification. The features were extracted using MobileNet-V2, and cataract severity was predicted with a random forest algorithm. The model attained an accuracy of 90.68% and a sensitivity of 91.43%. Gazala et al. [20] proposed a DenseNet-169 model for analysing fundus images to detect retinopathy and classify its severity. The images were processed, and the images were artificially increased and modelled, and the model attained an accuracy of 90%. Gahyung et al. [21] developed a fully automated classification for predicting diabetic retinopathy (DR) with the CNN model. Based on fluorescein angiography, salient facts of classification were verified. CNN attained an accuracy of 91% and a sensitivity of 86%. Mohamed et al. [22] proposed a hybrid inductive machine learning method (HIMLM) to process and diagnose fundus images as healthy or retinopathy. The images were improved by normalizing the images to a specific brightness level; the image was encoded and decoded for segmentation, feature extraction and classification using the HIMLM. The HIMLM attained an accuracy of 96.62% and a sensitivity of 95.31%. Veena et al. [23] proposed a framework for dividing the optic cup from the optic disc in order to find a ratio between them. The CNN model diagnosed glaucoma by segmenting the optic cup and optic disc to find a more efficient result. The model reached an accuracy of 97% and 98% for the segmentation of the optic cup and optic disc, respectively. Lamiaa et al. [24] developed a two-branched convolutional network to simultaneously extract anatomical features of blood vessels and optic discs. The network inputs use subband comparison and spatial retinal for wavelet approximation. The network attained an accuracy of 98.78% for spatial inputs and 96.34% for wavelet inputs. Jooyoung et al. [25] trained a deep-learning network on a fundus image dataset by narrowing the disc edge and increasing the cup-to-disc ratio. The patients’ eyes were interpreted using adversarial examples and Grad-CAM. Marriam et al. [26] developed the EfficientNet-B0 and EfficientDet-D0approach to overcome the similarity between lesions and eye colour by diagnosing glaucoma. Features were extracted from the fundus images with the EfficientNet-B0 extractor, and then EfficientDet-D0 features were calculated; the Bi-directional Feature Pyramid Network (BiFPN) module combined the features.

Rohit [27] proposed an intelligent system based on CNN and machine learning to analyse fundus images of colour and glaucoma. The hybrid system works in two stages: feature extraction with CNN and classification using machine learning algorithms. The logistic regression network outperformed other machine learning algorithms. Suchee et al. [28] presented three CNN models for predicting retinal diseases. The images were optimised with a Gaussian kernel and thresholded based on entropy to extract blood vessels. Rohit et al. [29] developed CNN, RF, SVM, and DT networks to classify a retinal dataset. The features were extracted and classified, and CNN excelled over the other networks.

There are still very few studies on the classification of eye diseases. Most studies focus on a specific type of eye disease and its stages of development. In this study, several systems were developed to classify eye diseases. Due to the similarity in eye diseases, especially at the beginning of the disease (early stages), it is difficult for ophthalmologists to distinguish between the types of disease. Therefore, this study focused on extracting accurate biological characteristics from the retina, the surrounding blood vessels, the optic cup, and the optic disc in order to distinguish the type of disease.

## 3. Methods and Materials

**Figure 1:**
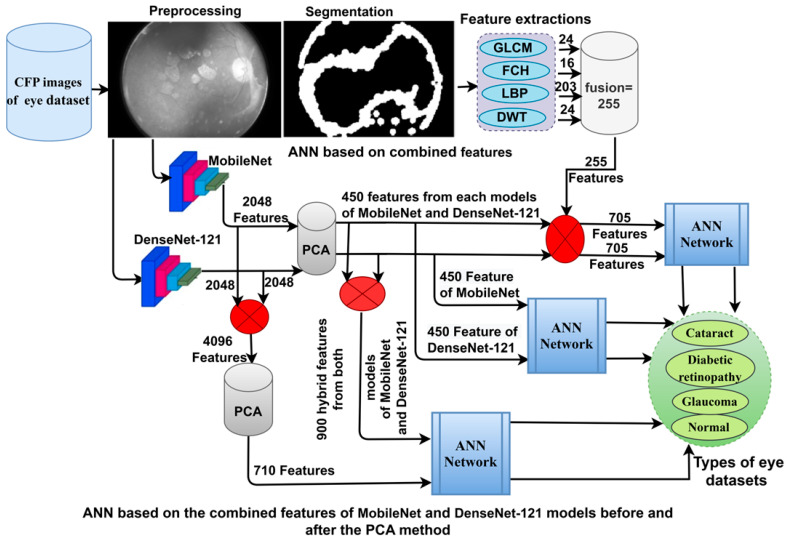
The basic structure of CFP image analysis methodologies for the classification of eye diseases.

This section uses techniques and materials to analyse CFP images for early classification of eye disease. The images were optimised and fed into three strategies, as shown in Figure 1.

### 3.1. Description of the Eye Disease Dataset

**Figure 2:**
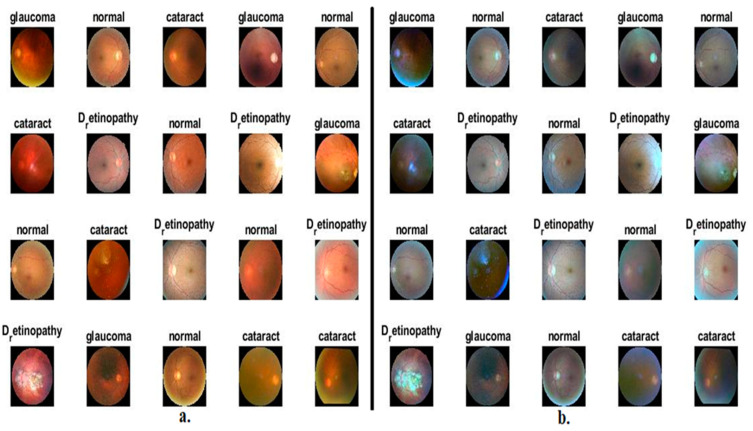
Examples of CFP pictures showing eye diseases (**a**) before improvement and (**b**) after improvement.

In this work, the performance of the proposed methods was assessed and generalised on CFP images for an eye disease dataset. The dataset images were collected from many sources such as Oculur Recognition, the Indian Diabetic Retinopathy Image Dataset (IDRiD), and High-Resolution Fundus (HRF), which consists of 4217 CFP images of three types of eye diseases and a normal class; this dataset is called the OIH dataset. The dataset contains nearly balanced classes; the cataract class contains 1038 images, and the diabetic_retinopathy class contains 1098 images. The glaucoma class contains 1007 images, and the normal class contains 1074 images. Figure 2a presents some sample images of CFP for the ophthalmic dataset [30].

### 3.2. Enhancement of the OIH Dataset Images

The presence of artefacts and low contrast in CFP images is a challenge for deep-learning models. These artifacts, such as eyelashes and their movement, and the difference in image-taking devices, lead to the collapse of deep-learning models and the lack of satisfactory accuracy. Furthermore, the low contrast between the optical cup and the optical disc and between the microvascular and their surroundings leads to a breakdown in the performance of the systems. Thus, this study applied an averaging filter to enhance CFP images for an eye disease dataset. The Laplacian filter increases the distinction of borders.

The average filter is used in image processing to smooth images and reduce noise. There are various ways to evaluate the performance of average filters. The Laplacian filter is an image filter commonly used in image processing to detect edges and features in images. There are various ways to evaluate the performance of Laplacian filters. Still, one objective numerical evaluation of the average and Laplacian filters is to use metrics such as the mean squared error (MSE) and edge preservation metrics.

These objective metrics provide numerical values that can be used to compare and evaluate different filters based on their performance, quality, or efficiency.

For the average filter, the MSE calculates the average squared difference between the pixel values of the filtered image and the original image. It provides a measure of the overall distortion caused by the filtering process. Lower MSE values indicate better image quality.

For the Laplacian filter, edge preservation metrics evaluate the filter’s ability to preserve edges or sharp transitions in the image. Examples include the Edge Preservation Index (EPI) and Structural Content (SC), which measure the similarity of edge maps between the original and filtered images. Higher values indicate better edge preservation.

An average filter of size 5 × 5 was passed over each CFP image in the eye disease dataset. Each time, the filter selected 25 pixels of the image and set a central pixel and 24 contiguous to it. The average of 24 contiguous pixels was computed and replaced [31]. The procedure is reiterated until each pixel in the image has been targeted, as shown in Equation (1).

(1) $$v\left(y\right)=\frac{1}{N}\sum_{i=0}^{N-1}z\left(y-i\right)\;\;$$

where *v* (*y*) is the enhanced CFP image, *z* (*y* − *i*) is the prior input, and *N* is the number of pixels.

After enhancing a fundus image, it is passed to a Laplacian to display the borders of the tiny blood vessels and the edges of the optic cup and the optic disc, as indicated in Equation (2).

(2) $$\nabla {\;}^{2}\;f=\frac{d\;f}{d^{\;2}\;x}+\frac{d{\;}^{2}\;f}{d^{\;2}\;y}$$

where *x* and *y* are the locations of the pixels.

Finally, the averaging filter enhancement is combined with the Laplacian image output, as in Equation (3).

(3) $$O\left(X\right)=v\left(y\right)-\nabla {\;}^{2}\;f$$

where $O\left(X\right)\;\mathrm{is}$ the enhanced image passed to the CNN models.

Figure 2b shows random CFP images from an eye disease dataset after enhancement.

### 3.3. Classification of CNN Features Using the ANN

**Figure 3:**
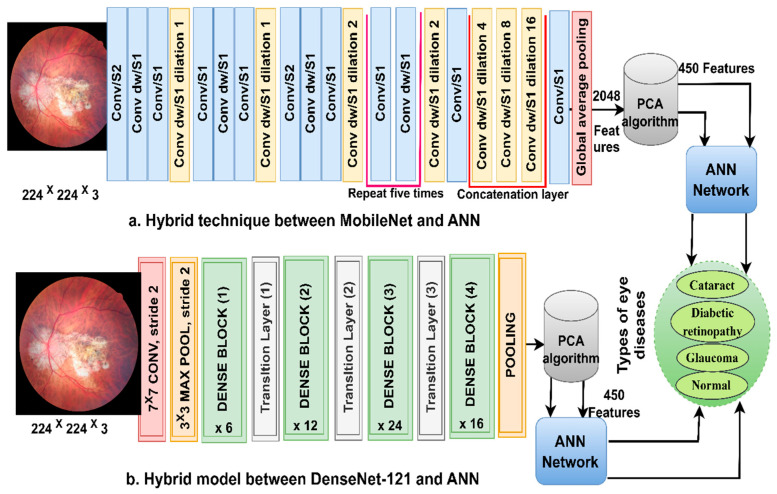
The basic structure of fundus image analysis for the classification of an eye disease dataset using ANN with MobileNet and DenseNet-121 features.

ANN-CNN is a hybrid technology with high efficiency in diagnosing biomedical images. In this study, this technique was used for several reasons, including its high efficiency in classifying CFP images for an eye disease dataset [32]. Furthermore, CNN creates millions of neurons, weights, biases, and connections; training a dataset on high-spec devices takes time, while hybrid technology is fast in training the dataset on low-cost devices. Hybrid technology is divided into operations, as displayed in Figure 3. First, the enhanced CFP images are fed to the MobileNet and DenseNet-121 models separately, which extract subtle and hidden features of eye disease CFP images using the convolutional layers (CL). The CL are among the important CNN layers; their number varies from one to another. Each layer serves a distinct function. Some layers work to display borders, whereas other layers extract colour characteristics, extract shape characteristics, and so on [33]. The size of the filter varies for each CL. The filter f(x) is convolved on the image x(t) to process it, and the procedure is replicated as in Equation (4). The size of the actual image is also maintained with the zero-padding parameter that works to pad the edges of the image with zeros. The filter is slid on the image with the p-step value. The CL generates millions of parameters, which makes the calculations more complex. Therefore, the pooling layer reduces the high dimensions using either max-pooling or average pooling. In the case of dimension reduction using the max-pooling layer, it selects a group of pixels, compares their values, and chooses a max value between them. Then, the group of pixel values is replaced with a max value between them, as in Equation (4). In the case of reducing the dimensions using the average-pooling layer, it selects a group of pixels and calculates their average [34]. Then, the set of pixel values is replaced with one value, as in Equation (5). The last convolutional layer produces features of size 4217 × 2048.

(4) $$y\left(i;\;j\right)=max_{m,n=1\ldots .v}\;f\left[\left(i-1\right)p+m;\;\left(\;j-1\right)p+n\right]$$

(5) $$y\left(i;\;j\right)=\frac{1}{v^{2}}\underset{m,n=1\ldots .v}{\sum }f\left[\left(i-1\right)p+m;\;\left(\;j-1\right)p+n\right]$$

where *m* and *n* denote the site in the matrix, *p* indicates the step of the filter, *v* denotes the features vectors, and *f* represents the filter.

Second, it is noticed that the size of the features is high and, thus, the features were fed to the PCA layer. PCA selects important features and eliminates unimportant and redundant features. PCA gets featured at 4217 × 450 from each model.

Third, the ANN divides the features into 80% for training and validation and 20% for testing. The network consists of three layers; the input layer has 450 input units to receive 450 features. There are fifteen hidden layers, which consist of neurons connected with the same layer and with other neurons’ layers. Information is passed from one layer to another, changing the weights linked to the neurons at each iteration [35]. The network computes the minimum square error (MSE) in each iteration of the expected $x_{i}$ and actual output $z_{i},$ as Equation (6). The work continues in the hidden layers until the network is stable and the MSE value does not change. The output layer from SoftMax labels each image and classifies it into an appropriate class. SoftMax generates four neurons as classes in the dataset, where each cell represents a class, and each image is categorised into a suitable class.

(6) $$\;MSE=\frac{1}{m}\overset{m}{\underset{i=1}{\sum }}\;{\left(\;x_{i}-z_{i}\right)}^{2}\;\;$$

where m denotes the input features, $x_{i}$ denotes the expected output, and $z_{i}$ denotes the actual output.

### 3.4. Classification of CNN-Fused Features Using the ANN

Here, we discuss the hybrid approach based on integrating features from two models, MobileNet and DenseNet-121, and their classification using an ANN [36]. This technique has been applied for several reasons, the most important of which is the representation of fundus images with very accurate features by merging the features from MobileNet with the DenseNet-121 model. The ANN also trains the dataset faster than CNN models with high classification accuracy.

In this section, CFP images from the eye disease dataset were classified using the feature fusion MobileNet and DenseNet-121 models. It is worth noting that the first method depends on extracting the features from DenseNet121 and MobileNet separately and then fusing the MobileNet and DenseNet-121 features. Then, the features are reduced with PCA and classified using the ANN [37]. The second method is based on using MobileNet and DenseNet121 separately for extracting the features and then decreasing the features separately with PCA separately. Then, the selected features are melted with PCA and sent to the ANN for classification.

**Figure 4:**
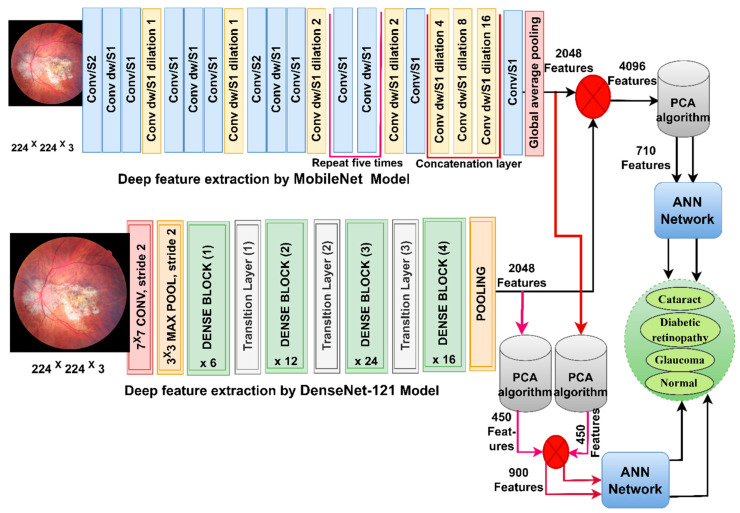
The basic structure of fundus image analysis for the classification of an eye disease dataset using ANN with fusion of the MobileNet and DenseNet-121 features.

Figure 4 shows the structure of the approach for categorising CFP images from an eye disease dataset using ANN according to the combined features before and after the MobileNet and DenseNet-121 feature dimensional reduction.

The first method goes through many steps as follows:

First, use enhancement filters to improve CFP pictures and identify the borders of the regions of interest. Second, extract subtle and hidden features in the CFP pictures using the convolutional and pooling layers for MobileNet and DenseNet-121 models separately and keep them at a size of 4217 × 2048 for each model. Third, fuse the MobileNet and DenseNet-121 features and keep them in a vector of size 4217 × 4096. Fourth, send high-dimensional feature vectors to the PCA to choose essential features, delete redundant and unimportant ones, and then save them in vectors of size 4217 × 710. Fifth, send the low dimensional feature vectors to the ANN and then divide them into 80% for network training and validation to measure its generalisability and 20% for system testing.

The second method to classify CFP images for an eye disease dataset goes through several steps as follows:

The first two steps are the same as in the first approach. Third, send the MobileNet feature to the PCA to choose essential features, delete repetitive and unimportant features, and then save them in vectors of size 4217 × 450. Fourth, send DenseNet-121 feature vectors to the PCA to choose essential features, delete repetitive and unimportant features, and then save them in vectors of size 4217 × 450. Fifth, fuse the MobileNet and DenseNet-121 features into a vector of size 4217 × 900. Sixth, feed the feature vectors to the ANN to divide them into 80% for network training and validation to measure its generalisation and 20% for system testing.

### 3.5. Classification of the Fusion between CNN and Handcrafted Features Using the ANN

**Figure 5:**
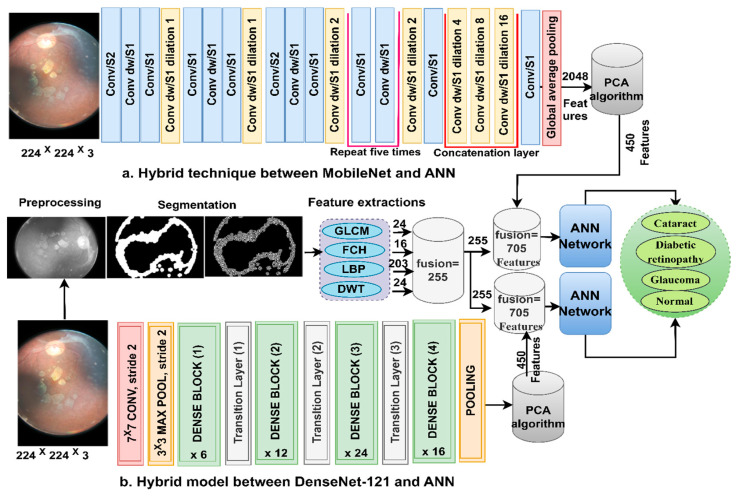
The basic structure of fundus image analysis for the classification of an eye disease dataset using ANN with the fusion of the CNN and handcrafted features.

Here, we discuss the hybrid approach based on combining CNN features (MobileNet and DenseNet-121) with handcrafted features (shape, texture, and colour) and their classification using an ANN. This technique is novel and has been applied to reach promising results by representing each image with high accuracy. The ANN trains the dataset faster than CNN models with high classification accuracy. The CFP images from the eye disease dataset were classified using two methods: first, based on the MobileNet and handcrafted features, and second, based on the DenseNet-121 and handcrafted features. Figure 5 illustrates the methodology structure for classifying the CFP images from an eye disease dataset using the ANN according to the combined features between deep learning (MobileNet and DenseNet-121) and conventional algorithms (GLCM, FCH, LBP, and DWT).

The first method goes through several steps as follows:

First, apply filters to improve the CFP pictures and the appearance of the borders in the regions of interest. Second, extract the important and hidden features of the CFP pictures using the CL of the MobileNet and DenseNet-121 models and keep them at a size of 4217 × 2048 for each model. Third, send the high-dimensional vectors to the PCA to identify the critical and unimportant features, and then keep them at a size of 4217 × 710 for each model separately. Fourth, extract the characteristics of shape, texture, and colour using the GLCM, FCH, LBP, and DWT methods [38].

GLCM displays the grey levels in the areas of interest. GLCM helps extract texture features to identify areas of difference in a region of interest. Smooth regions have similar pixel values, and rough regions have different pixel values, so statistical measures are useful for texture measurements. Thus, these measures depend on the spatial information on the spatial grey levels. The spatial information describes the association between pairs of pixels in terms of the distance d and the direction between a pixel and its neighbours θ (0°, 45°, 90°, and 135°) [39]. Thus, the method produces 24 statistical features for each CFP image of eye disease and saves them in vectors of size 4217 × 24.

Colour features are an important feature to help automatic systems make accurate diagnoses. Each colour in a region of interest is described in the histogram bin, each bin has similar colours even if they are different, and the two colours are different when they are in two different bins. The FCH method analyses the similitude of colours using the membership value per pixel and its diffusion on the histogram bin. Thus, this method produces 16 colour features for each CFP image of eye disease and saves them at a size of 4217 × 16.

LBP displays the grey levels in the areas of interest in a matrix. LBP helps characterise binary texture by calculating the local texture and disparity. The method assigns a size of 5 × 5 to each target pixel, and each time, the method selects a pixel and analyses it according to 24 adjacent pixels [40]. The method compares the grey levels of the central (g_c_) and adjacent (g_p_) pixels according to Equation (7). The method continues until each pixel has been analysed according to the equation. The method produces 203 features per CFP image of an eye lesion and saves them at a size of 4217 × 203.

(7) $$LBP_{R,P}=\overset{P-1}{\underset{p=0}{\sum }}s\left(g_{p}-g_{c}\right)2^{p}\;$$

where $g_{c}$ is the aim pixel, *P* represents the number of adjacent pixels, $g_{p}$ is the adjacent pixels, and *R* represents adjacent.

DWT analyses the CFP images of eye disease using quadrature mirror filters. The input signal is decomposed into two signals with different frequencies. The two signals correspond to the low and high filters. The two-dimensional image is analysed into four approximate and detailed components. Low filters serve to obtain approximate parameters, while the horizontal, diagonal, and vertical detail coefficients are obtained from the filter LH, HH, and HL, respectively. Thus, DWT analyses the binary signal into four bands for each level [41]. Each sub-band yields three features: mean, standard deviation, and variance. Therefore, this method produces 12 characters per CFP image of eye disease at a size of 4217 × 203.

In the fifth step, integrate the features of the traditional methods (GLCM, FCH, LBP, and DWT) and save them at a size of 4217 × 255.

Sixth, merge the MobileNet and handcrafted features at the size of 4217 × 705.

Seventh, merge the DenseNet-121 model and handcrafted features at the size of 4217 × 705.

## 4. Results of System Evaluation

### 4.1. Splitting of the Eye Disease Dataset

Several highly efficient systems have been developed to classify CFP images for eye disease datasets. The dataset was obtained from three sources called OIH, which consisted of 4217 CFP images divided between three types of eye diseases and a normal class. Table 1 displays the distribution of the OIH dataset within the training and validation (total of 80%) and testing phase (total of 20%).

### 4.2. Performance Evaluation Metrics for the Systems

Implementing the techniques on the two eye disease datasets was evaluated using the systems performance measures described in Equations (8)–(12). The confusion matrix is the golden output of system performance, which is a quadrilateral matrix that includes all data (images) that are correctly categorised as TN and TP and wrongly categorised as FN and FP. Thus, the data for the variables in the mentioned equations are provided from the data presented in the confusion matrix [42].

(8) $$\mathrm{AUC}\;=\frac{\mathrm{TP}\;\mathrm{Rate}}{\mathrm{FP}\;\mathrm{Rate}}$$

(9) $$\mathrm{Accuracy}=\frac{\mathrm{TN}+\mathrm{TP}}{\mathrm{TN}+\mathrm{TP}+\mathrm{FN}+\mathrm{FP}}\;*100\%\;$$

(10) $$\mathrm{Precision}=\frac{\mathrm{TP}}{\mathrm{TP}+\mathrm{FP}}\;*100\%$$

(11) $$\mathrm{Specificity}=\frac{\mathrm{TN}}{\mathrm{TN}+\mathrm{FP}}\;*100\;$$

(12) $$\mathrm{Sensitivity}=\frac{\mathrm{TP}}{\mathrm{TP}+\mathrm{FN}}\;*100\%$$

### 4.3. Data Augmentation

**Figure 6:**
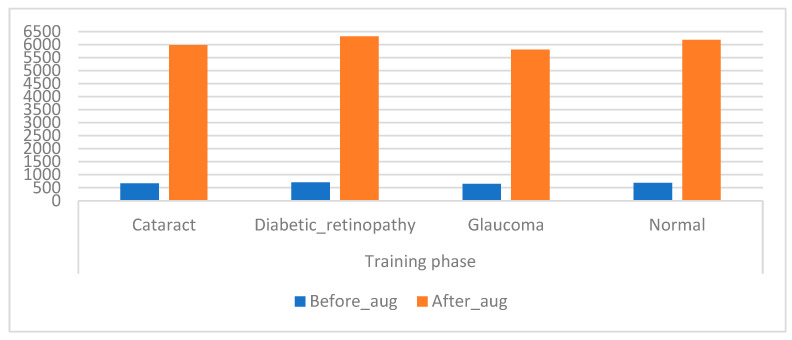
Displays showing the training dataset size before and after augmentation of the CFP images of eye disease.

CNN models have high capabilities in training huge datasets; the more data in the dataset, the better the CNN performance. Unfortunately, medical images are scarce, and there are privacy concerns when obtaining medical images from hospitals. Hence, this presents a challenge for CNN models, which causes an overfitting problem when using a CFP image from an eye disease dataset that lacks sufficient medical images to train CNN models [43]. This limitation has been solved by using the data augmentation process during the training phase. This method contains several operations to increase images from the same dataset artificially. The operations to increase images include flipping images, rotating them at several angles, shifting them, and others. Table 2 describes the training dataset from the original and after the increase in images. Figure 6 presents an illustration showing the increased images prior to and after data augmentation.

### 4.4. Results of CNN Features using ANN

The section describes the performance of the ANN, fed with the MobileNet and DenseNet-121 features separately. Because of the high-dimensional features from MobileNet and DenseNet-121, they were provided to PCA to reduce the dimensions by selecting important features and eliminating redundant features. After obtaining the important features only, they were sent to the ANN for division into 80% during the training of the systems and to adjust the network performance based on its error. The network was then validated based on its ability to adjust the generality, and the network training was stopped when the percentage of errors increased between the output and actual values and stopped circular on improving network performance. The remaining 20% was used as testing data to measure network performance.

**Figure 7:**
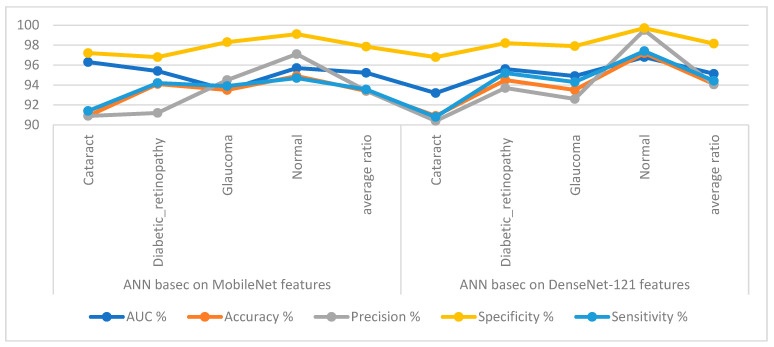
Display showing the ANN performance according to the MobileNet and DenseNet-121 features of the eye disease dataset classification.

Table 3 and Figure 7 display the implementation measurements of the ANN when the features of the MobileNet and DenseNet-121 models were classified separately. With the critical features from MobileNet, the ANN attained an AUC of 95.23%, an accuracy of 93.4%, a precision of 93.43%, a specificity of 97.85%, and a sensitivity of 93.55%. With the critical features from DenseNet-121, the ANN attained an AUC of 95.13%, an accuracy of 94.1%, a precision of 94.05%, a specificity of 98.15%, and a sensitivity of 94.43%.

**Figure 8:**
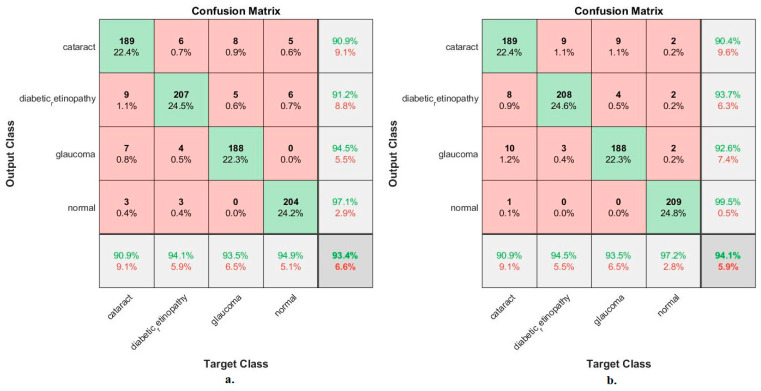
Confusion matrix used to measure the implementation of the ANN for classifying an eye disease dataset according to features from (**a**) MobileNet and (**b**) DenseNet-121.

Figure 8 shows the confusion matrix for measuring the ANN performance when classifying the features from MobileNet and DenseNet-121. With MobileNet features, the ANN achieved the following accuracies for all classes: 90.9% for cataracts, 94.1% for Diabetic_retinopathy, 93.5% for glaucoma, and 94.9% for the normal class. In contrast, with the DenseNet-121 features, the ANN achieved the following accuracies for all classes: 90.9% for cataracts, 94.5% for Diabetic_retinopathy, 93.5% for glaucoma, and 97.2% for the normal class.

### 4.5. Results of CNN-Fused Features using the ANN

This section discusses the ANN performance when provided with fused features from MobileNet and DenseNet-121. Due to the high-dimensional features from MobileNet and DenseNet-121, the dimensions were reduced with PCA before and after feature merging. Thus, the ANN was fed using two methods, MobileNet + DenseNet − PCA and MobileNet − PCA + DenseNet − PCA. After integrating the important features, they were sent to the ANN for division into 80% to train the systems and to adjust the network performance based on its error, and to validate the generalisation of the network. The remaining 20% was used as testing data to measure network performance.

**Figure 9:**
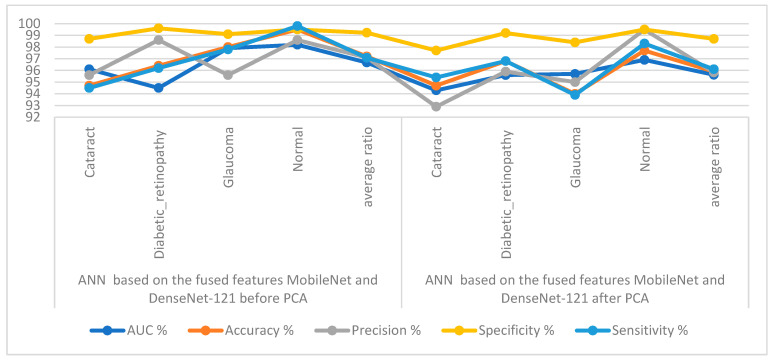
Display showing the ANN performance according to a combined feature from MobileNet and DenseNet121.

Table 4 and Figure 9 display the measurements of the ANN performance with fused features classified with the MobileNet and DenseNet-121 models. With the fused features from the MobileNet and DenseNet-121 models before dimension reduction, the ANN attained an AUC of 96.68%, an accuracy of 97.2%, a precision of 97.1%, a specificity of 99.23%, and a sensitivity of 97.08%. With the fused features from MobileNet and DenseNet-121, the ANN attained an AUC of 95.63%, an accuracy of 95.9%, a precision of 95.83%, a specificity of 98.7%, and a sensitivity of 96.1%.

**Figure 10:**
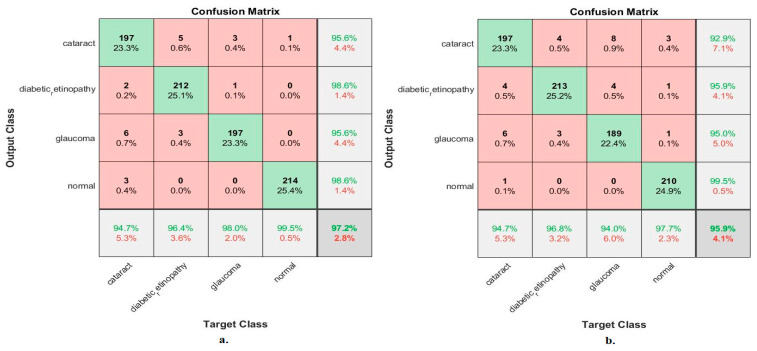
Confusion matrix measuring the ANN performance for classifying an eye disease dataset according to fused features from DenseNet121 and MobileNet (**a**) before using PCA and (**b**) after using PCA.

Figure 10 shows the confusion matrix for measuring the ANN performance when classifying fused features for the MobileNet and DenseNet-121 models. When fed with fused features from DenseNet-121 and MobileNet before dimension reduction, the ANN achieved the following accuracies for all classes: 94.7% for cataracts, 96.4% for diabetic retinopathy, 98% for glaucoma, and 99.5% for the normal class. In contrast, with the fused features from the Dense-Net-121 and MobileNet after dimension reduction, the ANN achieved the following accuracies for all classes: 94.7% for cataracts, 96.8% for diabetic retinopathy, 94% for glaucoma, and 97.7% for the normal class.

### 4.6. Results of the Fusion between CNN and Handcrafted Features Using the ANN

Here, we discuss the ANN implementation when provided with fused features from MobileNet with handcraft features and DenseNet-121 with handcraft features. Due to the high dimensional features of MobileNet and DenseNet-121, the high features were reduced with PCA before being combined with the handcrafted features. Hence, the ANN was fed using MobileNet-PCA with handcrafted features and DenseNet-PCA with handcrafted features. The features were sent to the ANN for division into 80% to train the systems and adjust the network performance based on its error, and then to validate the network’s generalisation and stop it from training. The remaining 20% was used as testing data to measure network performance.

**Figure 11:**
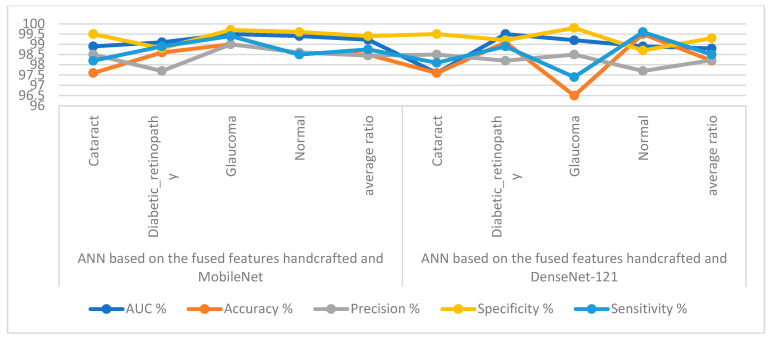
Display showing the ANN performance according to a combination of handcrafted and CNN features.

Table 5 and Figure 11 outline the ANN implementation measurements with features from MobileNet with handcraft features and DenseNet-121 with handcrafted features. With the MobileNet and handcrafted features, the ANN attained an AUC of 99.23%, an accuracy of 98.5%, a precision of 98.45%, a specificity of 99.4%, and a sensitivity of 98.75%. In contrast, with the DenseNet-121 and handcrafted features, the ANN attained an AUC of 98.8%, an accuracy of 98.2%, a precision of 98.23%, a specificity of 99.3%, and a sensitivity of 98.5%.

**Figure 12:**
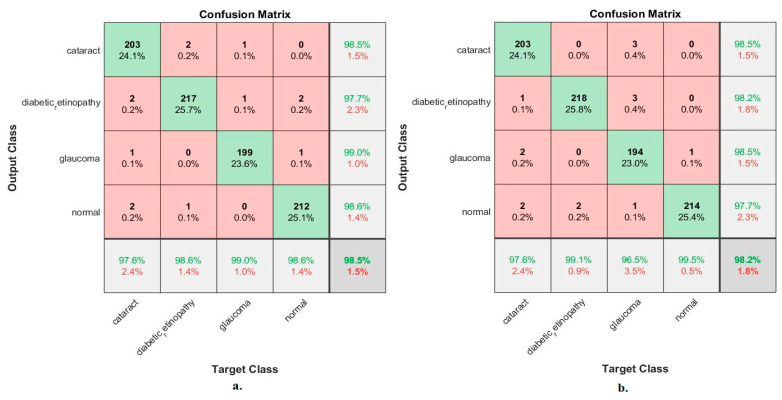
Confusion matrix for measuring the ANN performance for classifying an eye disease dataset according to fused (**a**) MobileNet and handcrafted features and (**b**) DenseNet-121 and handcrafted features.

Figure 12 illustrates the confusion matrix for measuring the ANN performance when classifying the CNN (MobileNet and DenseNet-121) and handcraft features. With the fused MobileNet and handcrafted features, the ANN attained the following accuracies for each class: 97.6% for cataracts, 98.6% for diabetic retinopathy, 99% for glaucoma, and 98.6% for the normal class. In contrast, with the fused DenseNet-121 and handcrafted features, the ANN attained the following accuracies for each category: 97.6% for cataracts, 99.1% for diabetic retinopathy, 96.5% for glaucoma, and 99.5% for the normal class.

This section also reviews some tools for measuring ANN performance with the fused features of MobileNet and DenseNet-121 and the handcrafted features as follows:

#### 4.6.1. Error Histogram

**Figure 13:**
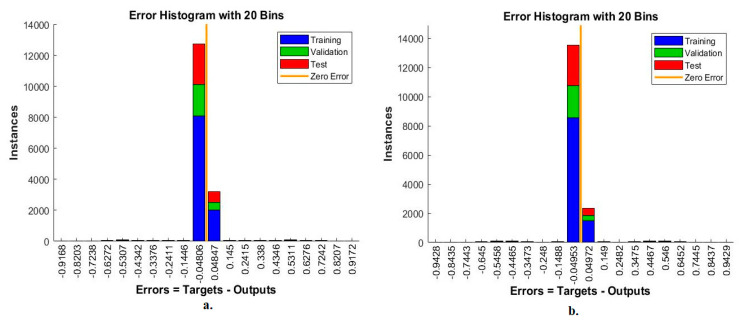
Error histogram for measuring the ANN performance for classifying an eye disease dataset according to the fused (**a**) MobileNet and handcrafted features and (**b**) DenseNet-121 and handcrafted features.

The error histogram is an ANN measurement tool for analysing CFP images to diagnose an eye disease dataset. The tool records the error in the target and the output during all stages according to the instances. The network records each stage’s performance in different colours, as in Figure 13. Red indicates the network measurement for the instances from the training phase, green indicates the network measurement for the instances from the validation phase, and blue indicates the network measurement for the instances from the testing phase [44]. It is observed that, with the MobileNet and handcrafted features, the ANN achieved the best execution between 20 bins within the values -0.9168 and 0.9172. In contrast, with the DenseNet-121 and handcrafted features, the ANN achieved the best execution between 20 bins within the values −0.9428 and 0.9429.

#### 4.6.2. Cross-Entropy

**Figure 14:**
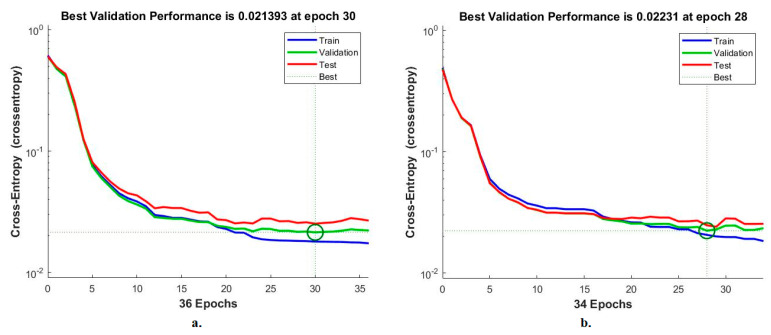
Cross-entropy for measuring the ANN performance for classifying an eye disease dataset according to the fused (**a**) MobileNet and handcrafted features and (**b**) DenseNet-121 and handcrafted features.

Cross-entropy is an ANN measurement tool for analysing CFP pictures to diagnose an eye disease dataset. The tool calculates the error in the target and the output in each period during all stages [42]. The network records each stage’s performance in different colours, as shown in Figure 14. The red colour shows the ANN measurement in the training stage, green indicates the ANN measurement in the validation stage, and blue indicates the ANN measurement in the testing stage. It is noted that the ANN, when fed with the MobileNet and handcrafted features, achieves superior performance with an error value of 0.021393 at epoch 30. In contrast, the ANN, when fed with the Dense-Net-121 and handcrafted features, achieves superior performance with an error value of 0.02231 at epoch 28.

#### 4.6.3. Gradient and Validation Checks

**Figure 15:**
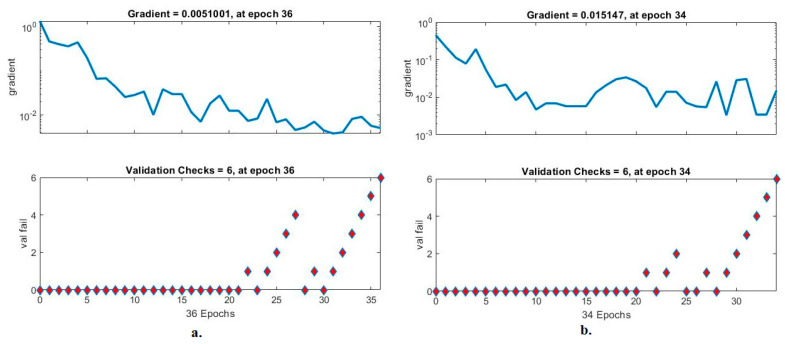
Gradient and validation checks for measuring the ANN performance for classifying an eye disease dataset according to the fused (**a**) MobileNet and handcrafted features and (**b**) DenseNet-121 and handcrafted features.

Validation checks are ANN measurement tool for analysing CFP images to diagnose an eye disease dataset. The tool checks the network performance hierarchy and finds failures during each epoch [45]. Figure 15 shows an ANN performance measure to check the performance gradient and failures with the eye disease dataset. With the fused handcrafted and MobileNet features, the ANN yielded a gradient of 0.0051001 at epoch 36 and a validation value of 6. With the fused handcrafted and DenseNet-121 model features, the ANN reached a gradient of 0.015147 at epoch 34 and a validation value of 6.

## 5. Discuss Performance Strategies

Eye diseases cause blindness if diagnosed late. Many eye diseases such as cataracts, diabetic retinopathy, and glaucoma have similar clinical symptoms and vital characteristics, especially in the early stages of the disease. Therefore, distinguishing between eye diseases is difficult and requires highly experienced doctors. In this work, three strategies were developed to classify CFP images from an eye disease dataset [46]. The CFP images of eye disease were enhanced with the same enhancement filters for all strategies. The overfitting problem was solved by overfitting the data.

The first strategy is to classify CFP images from the eye dataset by inserting optimised CFP images into the MobileNet and DenseNet-121 models separately. The two models analyse the images and extract all the hidden and accurate details from the images. Because the extracted data (features) are high-dimensional and contain redundant and non-significant features, the features from the MobileNet and DenseNet-121 models were fed into a PCA separately to choose the essential features. The ANN then receives the MobileNet-PCA and DenseNet-121-PCA features separately and trains and tests them. The ANN with MobileNet-PCA features achieved 93.4% accuracy, while with the features from Dense-Net-121-PCA, it attained an accuracy of 94.1%.

The second strategy is to classify CFP images from the eye dataset by inserting optimised CFP images into MobileNet and DenseNet-121 models separately. The two models analyse the images and extract the data for all the hidden and accurate details. This strategy consists of two systems according to combining features from the MobileNet and Dense-Net-121 models prior to and after high dimensionality reduction. In the first system of the second strategy, the features from MobileNet and DenseNet-121 are combined, and then the dimensions are reduced and fed to the ANN for classification. Based on the integrated features from MobileNet and DenseNet-121, the ANN attained an accuracy of 97.2%. In the second system of the second strategy, the features from DenseNet-121 and MobileNet are reduced and then fed to the ANN for classification. The ANN based on the combined features after dimension reduction of DenseNet-121 and MobileNet attained an accuracy of 95.9%.

The third strategy is to classify CFP images from the eye dataset by inserting optimised CFP images into DenseNet-121 and MobileNet models separately. The two models analyse the images and extract the data for all the hidden and accurate details. Because the extracted data (features) are high-dimensional and contain redundant and non-significant features, the features from the DenseNet-121 and MobileNet models were entered into the PCA separately to identify the significant features. This strategy consists of two systems. First, based on fusing the features from the MobileNet model and the handcrafted features. Second, based on fused DenseNet-121 and handcrafted features. Based on the fused MobileNet and handcrafted features, the ANN attained 98.5% accuracy. Based on fused DenseNet-121 and handcrafted features, the ANN attained 98.2% accuracy.

**Figure 16:**
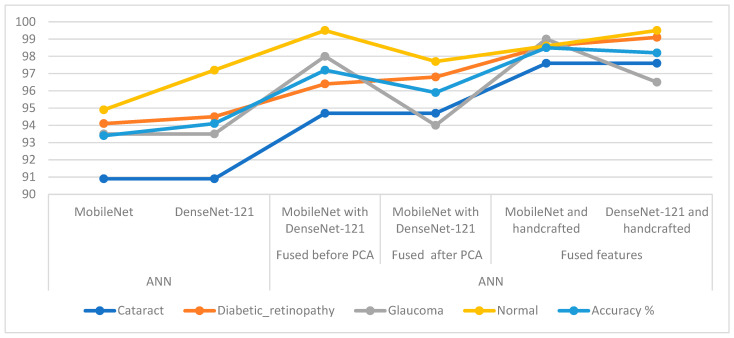
Display showing systems performance for classifying CFP images from an eye disease dataset.

In Table 6 and Figure 16, the results of the methods for classifying CFP pictures for the eye dataset are discussed. The table summarises accuracy in addition to each system’s accuracy at the class level. For the cataract class, the ANN reached the best accuracy with a percentage of 97.6% when provided with the fused CNN and handcrafted features. For the Diabetic_retinopathy class, the ANN attained an accuracy of 99.1% when provided with the fused DenseNet-121 and handcrafted features. For the glaucoma class, the ANN attained an accuracy of 99% when provided with fused MobileNet and handcrafted features. For the normal class, the ANN reached an accuracy of 99.5% when provided with fused handcrafted and Dense-Net-121 features in addition to providing the ANN with the fused features from MobileNet with DenseNet-121.

The results of the proposed systems were compared with the systems of previous studies on the IDRiD and HRF datasets used in this study. It is noted from Table 7 that the performance of the proposed system is superior to all systems compared to previous studies for all measures.

The limitation that we faced when implementing the systems was the lack of images in the dataset because deep-learning models need many images for the training process to generalise the system into any new dataset. This limitation was addressed by applying a data augmentation method to augment the dataset images and processing the dataset balancing in parallel.

The system has a superior ability to help doctors to distinguish between eye diseases with great accuracy and for the patient to receive the appropriate treatment according to their disease.

## 6. Conclusions

In this work, several systems were developed for multiple patient-level multiplex biomarker classification of CFP images from an eye disease dataset. There is a similarity in vital and clinical signs of eye diseases, especially in the early stages; therefore, the proposed systems focused on extracting all features, including hidden ones that are not visible to the naked eye, using many hybrid methods. This work discusses three strategies with six CFP image analysis regimes to classify an eye disease dataset. The first strategy is to classify eye diseases with an ANN based on the low dimensional features of two models, MobileNet-PCA and DenseNet-121-PCA. The second strategy for classifying eye diseases with an ANN is based on fused features from MobileNet and Dense-Net-121. It is worth noting that the fusion of features from MobileNet and DenseNet-121 is completed with two methods: before reducing dimensions and after reducing dimensions. The third strategy for classifying eye diseases with an ANN is based on the fused MobileNet and handcrafted features, and with an ANN based on the fused DenseNet-121 and handcrafted features. With the fused MobileNet and handcrafted features, the ANN attained an AUC of 99.23%, an accuracy of 98.5%, a precision of 98.45%, a specificity of 99.4%, and a sensitivity of 98.75%.

## Figures and Tables

**Table 1:** Splitting of the CFP images of eye diseases.

Phase	80:20%		20% for Testing
Classes	80% for Training	20% for Validation	
Cataract	664	166	208
Diabetic_retinopathy	702	176	220
Glaucoma	645	161	201
Normal	687	172	215

**Table 2:** Augmented images of the eye disease dataset.

Phase	Training Phase			
Classes	Cataract	Diabetic_Retinopathy	Glaucoma	Normal
Before_aug	664	702	645	687
After_aug	**5976**	**6318**	**5805**	**6183**

**Table 3:** ANN results with MobileNet and DenseNet-121 features.

Methods	Classes of AD	AUC%	Accuracy%	Precision%	Specificity%	Sensitivity%
ANN based on MobileNet features	Cataract	96.3	90.9	90.9	97.2	91.4
	Diabetic_retinopathy	95.4	94.1	91.2	96.8	94.2
	Glaucoma	93.5	93.5	94.5	98.3	93.9
	Normal	95.7	94.9	97.1	99.1	94.7
	**average ratio**	**95.23**	**93.4**	**93.43**	**97.85**	**93.55**
ANN based on DenseNet-121 features	Cataract	93.2	90.9	90.4	96.8	90.8
	Diabetic_retinopathy	95.6	94.5	93.7	98.2	95.2
	Glaucoma	94.9	93.5	92.6	97.9	94.3
	Normal	96.8	97.2	99.5	99.7	97.4
	**average ratio**	**95.13**	**94.1**	**94.05**	**98.15**	**94.43**

**Table 4:** ANN results with MobileNet and DenseNet-121 fused features.

Methods	Classes of AD	AUC%	Accuracy%	Precision%	Specificity%	Sensitivity%
ANN based on the fused features MobileNet and DenseNet-121 before PCA	Cataract	96.1	94.7	95.6	98.7	94.5
	Diabetic_retinopathy	94.5	96.4	98.6	99.6	96.2
	Glaucoma	97.9	98	95.6	99.1	97.8
	Normal	98.2	99.5	98.6	99.5	99.8
	**average ratio**	**96.68**	**97.2**	**97.10**	**99.23**	**97.08**
ANN based on the fused features MobileNet and DenseNet-121 after PCA	Cataract	94.3	94.7	92.9	97.7	95.4
	Diabetic_retinopathy	95.6	96.8	95.9	99.2	96.8
	Glaucoma	95.7	94	95	98.4	93.9
	Normal	96.9	97.7	99.5	99.5	98.3
	**average ratio**	**95.63**	**95.9**	**95.83**	**98.70**	**96.10**

**Table 5:** ANN Results with fused features handcrafted and CNN.

Methods	Classes of AD	AUC%	Accuracy%	Precision%	Specificity%	Sensitivity%
ANN based on the fused features handcrafted and MobileNet	Cataract	98.9	97.6	98.5	99.5	98.2
	Diabetic_retinopathy	99.1	98.6	97.7	98.8	98.9
	Glaucoma	99.5	99	99	99.7	99.4
	Normal	99.4	98.6	98.6	99.6	98.5
	**average ratio**	**99.23**	**98.5**	**98.45**	**99.40**	**98.75**
ANN based on the fused features handcrafted and DenseNet-121	Cataract	97.6	97.6	98.5	99.5	98.1
	Diabetic_retinopathy	99.5	99.1	98.2	99.2	98.9
	Glaucoma	99.2	96.5	98.5	99.8	97.4
	Normal	98.9	99.5	97.7	98.7	99.6
	**average ratio**	**98.80**	**98.2**	**98.23**	**99.30**	**98.50**

**Table 6:** Summary of systems results for CFP classification of the eye disease dataset.

Techniques		Features	Cataract	Diabetic_Retinopathy	Glaucoma	Normal	Accuracy%
ANN		MobileNet	90.9	94.1	93.5	94.9	93.4
		DenseNet-121	90.9	94.5	93.5	97.2	94.1
ANN	Fused before PCA	MobileNet with DenseNet-121	94.7	96.4	98	99.5	97.2
	Fused after PCA	MobileNet with DenseNet-121	94.7	96.8	94	97.7	95.9
	Fused features	MobileNet and handcrafted	97.6	98.6	99	98.6	98.5
		DenseNet-121 and handcrafted	97.6	99.1	96.5	99.5	98.2

**Table 7:** Comparison of the performance of the systems with the performance of the systems from previous studies.

Previous Studies	Accuracy%	AUC%	Sensitivity%	Precision%	Specificity%
Liu et al. [47]	86.70	-	75.60	-	77.80
Sundaram et al. [48]	95.28	-	94.1	-	95.34
Bilalet al. [49]	94.54	-	87.09	-	95.4
Junayed et al. [16]	95.02	-	95.68	94.86	94.79
Gayathriet al. [50]	-	-	97.8	92.9	96.4
Zhan et al. [51]	56.19	-	64.21	-	87.39
Saranyaet al [52]	95.65	-	89	-	99
Bhardwaj et al. [53]	92.39	-	84.21	-	93.48
**Proposed model**	**98.5**	**99.23**	**98.75**	**98.45**	**99.4**

## Data Availability

The CFP images of ocular diseases that supported the performance measurement of the systems were obtained from a publicly available dataset at: https://www.kaggle.com/datasets/gunavenkatdoddi/eye-diseases-classification (accessed on 12 October 2022).
